# The Relationship between Depressive Symptoms, Disease State, and Cognition in Amyotrophic Lateral Sclerosis

**DOI:** 10.3389/fpsyg.2012.00542

**Published:** 2012-12-17

**Authors:** Laura Jelsone-Swain, Carol Persad, Kristen L. Votruba, Sara L. Weisenbach, Timothy Johnson, Kirsten L. Gruis, Robert C. Welsh

**Affiliations:** ^1^Department of Radiology, University of MichiganAnn Arbor, MI, USA; ^2^Department of Psychiatry, University of MichiganAnn Arbor, MI, USA; ^3^Department of Biostatistics, University of MichiganAnn Arbor, MI, USA; ^4^Department of Neurology, The State University of New York Upstate Medical UniversitySyracuse, NY, USA

**Keywords:** ALS, motor neuron disease, neuropsychology, depression, ALSFRS-r, cognition, limb symptoms

## Abstract

Cognitive impairment (CI) in amyotrophic lateral sclerosis (ALS) may present a serious barrier to a patient’s wellbeing and significantly decrease quality of life. Although reports of CI in ALS without frank dementia are becoming quite common, questions remain regarding the specific cognitive domains affected, as well as how other psychological and medical factors may impact cognitive functioning in these patients. Additionally, the influence of depressive symptoms on disease processes is not known. We aimed to address these questions by completing extensive neuropsychological tests with 22 patients with ALS and 17 healthy volunteers. A subgroup of these patients also completed questionnaires to measure depressive and vegetative symptoms. We tested for overall cognitive differences between groups, the influence of physical (e.g., bulbar and limb), vegetative (e.g., fatigue), and depressive symptoms on cognitive performance, and the relationship between depressive symptoms and disease severity in ALS. Overall, patients performed more poorly than healthy controls (HCs), most notably on tests of executive functioning and learning and memory. Results suggest that true cognitive performance differences exist between patients with ALS and HCs, as these differences were not changed by the presence of vegetative or depressive symptoms. There was no effect of limb or bulbar symptoms on cognitive functioning. Also, patients were not any more depressed than HCs, however increased depressive scores correlated with faster disease progression and decreased limb function. Collectively, it is suggested that translational advances in psychological intervention for those with CI and depression become emphasized in future research.

## Introduction

Until recently, amyotrophic lateral sclerosis (ALS) was still considered by many a pure motor neuron disease (Pongratz, 2000), and the notion of cognitive impairment (CI) in ALS was met with much resistance until just the past several years (Bak and Chandran, 2012). It is now widely accepted that ALS is a multisystem disorder involving CI in approximately 30% of those diagnosed with the disease (Rippon et al., 2006). ALS with CI is now recognized as a subcategory of ALS (Strong et al., 2009) and is denoted as ALS*ci* (Murphy et al., 2007), which entails presentation of CI without frank dementia. Importantly, some experts propose that ALS is not a categorical disease, but rather a spectrum disorder between pure ALS and Frontotemporal Lobar Degenerative diseases, such as Frontotemporal dementia (Abrahams et al., 2000; Grossman et al., 2007; Murphy et al., 2007; Woolley and Katz, 2008; Merrilees et al., 2010).

Recent advances in the field support the need for study into cognitive problems associated with ALS (see Phukan et al., 2007). One critical reason for this research is that cognitive difficulties in patients with ALS negatively impact quality of life, and appear to be even more detrimental to quality of life than physical impairment (Goldstein et al., 2002). Personal communication with patients who believe their cognitive ability has been compromised report being negatively impacted by these symptoms and wanting help, or at least support, in this area. Additionally, CI can influence decision-making (Girardi et al., 2011), treatment compliance (Olney et al., 2005), survival (Olney et al., 2005; Flaherty-Craig et al., 2011), and even the wellbeing of caregivers (Merrilees et al., 2010).

Most studies that have identified CI in patients with ALS have utilized a comprehensive neuropsychological battery to assess performance across multiple modalities. Of these studies, verbal fluency remains one of the most sensitive measures for CI, repeatedly showing poorer performance in those with ALS compared to healthy individuals (Abrahams et al., 1997, 2000; Kilani et al., 2004; Grossman et al., 2007). These findings hold even after correcting for possible verbal production impairment (Abrahams et al., 2004). Other studies have found decreased performance in other areas of executive function (Kilani et al., 2004), memory (Abrahams et al., 1997; Hanagasi et al., 2002), and language (Hillis et al., 2004; Raaphorst et al., 2010). Although reports of CI in ALS are becoming more common, characteristics of ALS*ci* are not well defined due to a number of factors. These include the lack of availability of valid cognitive measures that can be administered to ALS patients with significant motor or speech limitations, as well as minimal research focusing on additional variables that could also impact cognition, such as physical symptoms and mood state.

As previously mentioned, ALS*ci* is not well understood, and the involvement of physical symptoms on CI in ALS is not known. Specifically, previous research has indicated that those with bulbar onset are more likely to have ALS*ci* (Gordon et al., 2011), yet others have not supported this notion (Rusina et al., 2010). Additionally, vegetative physical symptoms of ALS may impact the interpretation of those identified with ALS*ci*. For example, it is known that fatigue, one of the most common vegetative symptoms in ALS, interferes with cognitive test performance in other patient groups (Diamond et al., 2008). For the purpose of this paper, vegetative symptoms collectively refer to changes in concentration, fatigue, appetite, sleeping, and energy level. Although these symptoms can also be specific to depression, ALS patients with physical weakness are likely to have more vegetative symptoms in the performance of daily activities rather than from depression, especially if the patient does not report other symptoms of depression such as hopelessness and guilt. Therefore, it is necessary to differentiate between physical and depressive symptoms in predicting CI in order to obtain a more accurate description of CI in patients with ALS. This may also lead to more streamlined classification of those with true CI who may need or want intervention.

Like CI, research suggests that depressive symptoms in ALS may also have a negative impact on many aspects of quality of life (Tramonti et al., 2012), and could potentially affect cognition. To our knowledge however, no studies have examined the influence of depressive symptoms on cognitive performance in ALS. Like vegetative symptoms, mood status could present a potential confound in the diagnosis of ALS*ci*. Also, depressive symptoms may increase the risk of having CI, or differentially affect cognition in patients with ALS compared to a healthy control population. These situations are likely, as depression can negatively influence cognitive performance in other diseases (Diamond et al., 2008; Sassoon et al., 2012).

The relationship between depression and prognosis or physical symptom presentation in ALS is another area of research that has received little attention, possibly due to the general report of a global positive psychosocial adjustment in these patients (Brown and Mueller, 1970; Lulé et al., 2012). Despite this view, prevalence rates of depression (McLeod and Clarke, 2007) and reports on the effect of depression on ALS are not consistent. For example, Atassi et al. (2011) reported no relationship between disease progression and depression, yet a previous study identified a significant negative impact of psychological distress on mortality outcome (McDonald et al., 1994). Using the ALS Functional Rating Scale (ALSFRS), a group in South Korea found that decreased physical functioning correlated with depressive symptoms (Oh et al., 2012), highlighting the importance of examining this relationship. Patients in this study however were on average both highly physically disabled (ALSFRS mean score of 19) and many were clinically depressed (Beck Depression Inventory mean score of 24.5). If consistent evidence supports the association of depressive symptoms with disease processes (especially while patients are still early in the disease course and without clinical depression), this would bring about new emphasis into the study of depression in ALS, ultimately leading to improved identification of those at highest risk and thus early intervention.

The overall goal of this study was to recognize if cognitive status is affected in patients with ALS early in their disease course and without severe clinical depression. We also examined other variables that may be associated with cognitive performance that could either facilitate in future early screening or confound the diagnosis of ALS*ci*. Specifically, we investigated the relationship of physical impairment, vegetative symptoms, and depressive symptoms on cognitive functioning. In addition, we tested whether depressive symptoms are associated with disease progression or physical impairment. We utilized standard neuropsychological tests to measure these relationships. Based on previous literature, we hypothesized that patients with ALS would perform at a lower level on tests within the neuropsychological battery, especially those targeting executive functioning skills. We predicted that physical, vegetative, and depressive symptoms would negatively influence cognitive performance, but that these would not be confounding variables in performance differences between patients with ALS and healthy volunteers (suggesting true cognitive impairment in the ALS group). We also hypothesized that ALS patients would have more depressive symptoms than healthy controls, and that increased depressive symptoms would be related to disease severity.

## Materials and Methods

### Participants

Twenty-two patients with ALS participated in this study. All patients were recruited through the ALS Clinic at the University of Michigan. Patients were diagnosed by a neuromuscular physician at the University of Michigan using the El Escorial criteria for ALS. Enrolled patients were engaged in a larger longitudinal neuroimaging component of this study; therefore all patients were without any contraindications to magnetic resonance imaging (MRI), were able to lay flat on their back without respiratory distress, were not dependent on artificial ventilation, and were ambulatory. Twenty-three healthy controls (HCs) were recruited through the general community from an internet-based recruitment tool (UMClinicalTrials.org) and from flyers posted around the Ann Arbor, MI area. Participants were excluded from either group if they had a history of alcohol or drug abuse, neurological disease (other than ALS), were ever diagnosed with a psychological disorder, or were severely clinically depressed at the time of their participation. No patient was excluded from the study, and one healthy volunteer was excluded for major depression. Because we aimed to compare cognitive status in patients with ALS to a truly healthy control group, HCs at risk for mild CI (see Statistical Analysis) were also excluded from the final analysis. Participant demographic information for these final groups is displayed in Table 1. Signed consent from each participant was obtained, and all aspects of this study were approved by the Institutional Review Board at the University of Michigan.

**Table 1:** **Means and SD for demographic information by group**.

Demographic variables	ALS patients	Healthy controls
Age	59 (6.6)	58 (5.0)
DH	18R*, 4L	15R, 2L
Sex	13M, 9F	5M, 12F
Education years	13.4 (2.0)	16 (2.6)
Symptom onset	17L, 5B	NA
ALSFRS-r total	37.2 (6.9)	NA
ALSFRS-r limb	16.5 (5.3)	NA
ALSFRS-r bulbar	10.0 (2.4)	NA
ALSFRS-r resp.	10.73 (1.9)	NA
MSO	26.2 (35.6)	NA

DH, dominant hand; Education Years, number of complete years of education, considering degrees obtained; ALSFRS-r Limb, subscore from ALSFRS-r questionnaire assessing limb function (questions 4–9); ALSFRS-r Bulbar, subscore from ALSFRS-r questionnaire assessing bulbar function (questions 1 – 3); ALSFRS-r Resp., subscore from ALSFRS-r questionnaire assessing respiratory function (questions 10 – 12); MSO, months since symptom onset. *Two patients reported current dominant hand as a result of disease processes and not by preference.

### Neuropsychological assessments

All participants completed a 60-min neuropsychological battery designed by a team of neuropsychologists experienced in motor diseases (lead by CP). These tests were selected to assess a broad range of cognitive skills, particularly those commonly associated with frontotemporal dementia, given the reported prevalence of such deficits in patients with ALS. Depressive and vegetative symptoms were also measured using the Geriatric Depression Scale, short version (GDS), and the Beck Depression Inventory (BDI), respectively. Not all participants completed both questionnaires. Nineteen ALS patients and 15 HCs completed the GDS, and 14 patients and 15 HCs completed the BDI. All tests and questionnaires are described below. Reliability coefficients and, when available, construct validity coefficients are given for each measurement (Strauss et al., 2006).

#### Mini-Mental State Examination (Folstein et al., 1975)

The Mini-Mental State Examination (MMSE) is a brief test of global cognitive function and is often used as a screen for dementia. The scale assesses a subject’s orientation to time and place, instantaneous recall, short-term memory, and ability to perform serial subtractions or reverse spelling. The MMSE also measures constructional capacities (the ability to copy a design) and the use of language. Internal consistency of this test ranges between 0.31–0.96, test re-test reliability ranges between 0.80–0.95, and construct validity is modest to high in correlating with other similar tests.

#### Hopkins Verbal Learning Test (Shapiro et al., 1999)

The Hopkins Verbal Learning Test (HVLT) is a learning and memory test that measures initial recall (test re-test reliability 0.74), delayed recall (20 min, test re-test reliability 0.66), and discrimination recall (test re-test reliability 0.40) for a list of words read by the administrator. The test is available in multiple alternate forms with a moderate to high construct validity with other similar tests.

#### Faces Subtest: Wechsler Memory Scale (WMS-III; subtests – Faces) (Wechsler, 1997)

Faces is a test of visual learning and recognition memory for pictures of faces. There is an initial recall test (Faces I) and a delayed recall test (Faces II, 30 min after first exposure to the set of faces). Internal reliability for these tests is between 0.60–0.96, internal consistency is between 0.70–0.79, and construct validity is modest.

#### Boston Diagnostic Aphasia Examination: Complex Ideational Material (Goodglass and Kaplan, 1983)

The Complex Ideational Material (CIM) subtest of the Boston Diagnostic Aphasia Examination (BDAE) measures auditory comprehension. This task requires patients to understand and express agreement or disagreement concerning factual material that are derived from inference. Complex Ideational Material has an internal consistency of 0.80 and a construct validity score between 0.86 and 0.93. This test was originally designed for patients with aphasia, so test-re-test reliability measurements were not provided as test performance is expected to change with this group.

#### The Boston Naming Test (Kaplan et al., 1978)

The Boston Naming Test (BNT) is a confrontation-naming test that is largely used to measure language processing. The test consists of 60 large pen and ink drawings of items ranging in familiarity. Participants are asked to freely name each item. If the identity of an item is not known, first a stimulus cue is provided, then a phonemic cue if needed. Scores used in this study only include freely identified and stimulus-cued correct responses. Internal consistency coefficients range between 0.78–0.96 and test re-test reliability is high (0.91). Construct validity is also high.

#### Judgment of Line Orientation (Benton, 1983)

The Judgment of Line Orientation (JOLO) measures visuospatial ability, specifically the ability to estimate angular relationships between line segments by visually matching angled line pairs to numbered radii that form a semicircle. The internal consistency for this test is between 0.84 and 0.91 and the test re-test coefficient is 0.90. Construct validity is high when compared with other visual spatial subtests.

#### Digit Span (Wechsler Adult Intelligence Scale: WAIS subtest) (Wechsler, 1997)

Digit Span is a subtest from the WAIS measuring auditory working memory. This test consists of two sections measuring immediate recall of a forward and backward digit series. In this study the total cumulative score of number of correct trials was used to measure overall working memory recall. Backward digit span is more challenging and was also used as a measurement in this study. The maximum span of backward digits recalled was used; therefore this measurement was not collinear with the total cumulative score. The generalizeability coefficient for this test is high (0.80–0.89) and the test re-test coefficient for the backward subscore is marginal (0.60–0.69).

#### Oral Trail-Making Test (Lezak, 1995)

The Oral Trail-Making Tests (OTMT) are tests of processing speed (Trails A) and conceptual ability (Trails B). For Trails A, the subject is required to recite consecutive numbers, from 1–25, as quickly as possible. On Trails B, subjects are asked to alternate between numbers and letters, from 1-A to 13-M. Two scores for each part are obtained – the total time and number of errors. To more accurately measure set-shifting abilities from Oral Trail-Making Test B and partial out motor speed due to possible verbal speed production impairment in patients with ALS (Abrahams et al., 2000), time (in seconds) to complete Oral Trail-Making Test B was divided by time (in seconds) to complete Oral Trail-Making Test A, resulting in an Oral Trails B/Oral Trails A ratio. This ratio results in a more statistically accurate measure to account for processing speed differences between groups. The test re-test reliability is adequate for test A and high for test B. Tests A and B correlate well with each other (0.31).

#### Controlled Oral Word Association Test (Benton, 1983)

The Controlled Oral Word Association Test (COWAT) is a test of verbal letter fluency using the letters C, F, and L. Participants are asked to name as many words beginning with each letter in 1 min (each). This is largely a test of executive function. The internal consistency for this test, specifically using the letters CFL, is high (0.83). Test re-test reliability is high (0.70) and construct validity is high.

#### Animal Naming (Sager et al., 2006)

The Animal Naming test is a categorical verbal fluency test and is used as a test of executive function. Participants are asked to name as many animals as they can in 1 min. Test re-test reliability is 0.56 and construct validity is moderately high.

#### Geriatric Depression Scale-Short Form (Yesavage and Sheikh, 1986)

This 15-(yes/no) questionnaire was developed specifically to measure depression in older adults. Symptoms common to normal aging and those pertaining to feelings of guilt, sexual activity, suicide, and somatic symptoms are not included in this questionnaire. Rather, this questionnaire focuses on the individual’s locus of control, “… making this more suitable for subjects in hospital or long-term care” (Strauss et al., 2006, p. 1104). Therefore the Geriatric depression scale-short form (GDS) was deemed as a more valid measure of core depressive symptoms in the ALS group and was used as a measure of depression severity. The short version has a high correlation with the full version, which is moderately to highly correlated with other tests of depression (0.73–0.91). The internal consistency for the full version ranges between 0.71 and 0.84 and test re-test reliability is 0.84.

#### Beck Depression Inventory (Beck et al., 1996)

This 21-item likert-scale questionnaire is a brief self-report of depression. The BDI contains a number of somatic and vegetative symptoms that can overlap with physical illness, more common in older adults and especially those with motor neuron diseases. Therefore impetus for completing the BDI was to provide a measure of vegetative symptoms to asses show they may contribute to cognitive functioning. Five questions were selected to describe vegetative symptoms: 15. Loss of Energy; 16. Changes in Sleeping Pattern; 18. Changes in Appetite; 19. Concentration Difficulty; 20. Tiredness or Fatigue. The internal consistency coefficient for this questionnaire is high (0.88) and the test re-test reliability is adequate to high (0.74–0.93). The construct validity is also high.

### Statistical analysis

Neuropsychological test results used to compare cognitive performance between groups are listed in Table 2. All data is presented in raw scores. All data were examined to check for parametric analysis statistical assumptions. Results from two tests did not meet these assumptions: the Complex Ideational Material (CIM) and Oral Trails-Making Test B/A ratio scores. In the CIM, the majority of participants performed at ceiling level (maximum score of 12 points), with just one person scoring nine points. The CIM was therefore excluded from further statistical models. The Oral Trails B/A ratio score was slightly positively skewed in both groups. Therefore individual scores from the Oral Trails B/A ratio were transformed (using the natural logarithm function), which resulted in a normal distribution for both groups. IBM’s statistical software package SPSS, version 19, was used to conduct all analyses.

**Table 2:** **Neuropsychological test results between patients with ALS and healthy controls**.

Cognitive domain	Neuropsychological test measurement	ALS patients (*n* = 22)		Healthy controls (*n* = 17)		Group differences	
		Mean raw score (SD)	% Impaired	Mean raw score (SD)	% Impaired	*F*-value	*p*-value
Mental status/dementia screen	MMSE total score	27.41 (2.04)	na	27.65 (1.50)	na	0.31	0.58
Executive function	COWAT (CFL) total score	32.77 (9.25)	4.50	49.53 (12.62)	0.0	11.12	<0.01
	Animal Naming total score	15.91 (4.22)	36.36	22.06 (4.59)	5.8	10.04	<0.01
	OTMT B seconds	44.68 (29.62)	4.50	25.56 (15.49)	0.0	na	na
	OTMT transformed ratio (B seconds/A seconds)	1.32 (0.55)	na	1.18 (0.39)	na	1.19	0.28
Memory/learning	HVLT initial recall, trials 1–3	23.86 (5.82)	31.80	27.24 (4.13)	5.8	1.10	0.30
	HVLT delayed recall, trial 4	8.59 (2.40)	27.3	9.94 (1.39)	0.0	1.65	0.21
	HVLT discrimination score	10.18 (1.62)	18.2	11.24 (0.97)	0.0	2.68	0.11
	Faces I total score	34.86 (4.81)	4.5	39.00 (4.47)	0.0	10.390	<0.01
	Faces II total score	37.05 (4.87)	9.0	40.65 (2.78)	0.0	6.81	0.01
Attention/concentration	Digit Span total score	15.68 (3.72)	4.5	18.12 (3.33)	0.0	2.53	0.12
	Digit Span backward span	4.50 (1.06)	0.0	5.24 (1.35)	0.0	0.83	0.37
Visuoperceptual	JOLO total score	23.91 (5.38)	13.63	25.88 (4.26)	5.8	0.05	0.83
Language	BNT total score including semantic cues	56.55 (2.46)	0.0	55.94 (3.15)	0.0	3.95	0.06
Depression	GDS (ALS *n* = 19, HC *n* = 15)	2.32 (1.97)	na	1.07 (1.16)	na	(*t*) 2.17	0.04
	BDI (ALS *n* = 14, HC *n* = 15)	5.57 (3.80)	na	3.00 (2.17)	na	(*t*) 2.26	0.03
Vegetative symptoms	BDI_veg (ALS *n* = 14, HC *n* = 15)	2.64 (1.74)	na	1.67 (1.29)	na	(*t*) 1.73	0.10

*MMSE, Mini-Mental State Exam; COWAT, Controlled Oral Word Association Test; OTMT, Oral Trail-Making Test; HVLT, Hopkins Verbal Learning Test; Faces I & II from the Wechsler Memory Scale III; JOLO, Judgment of Line Orientation; BNT, Boston Naming Test; GDS, Geriatric Depression Scale; BDI, Beck Depression Inventory; BDI_veg, five questions measuring vegetative symptoms taken from the BDI; % impaired, percent of participants in each group with a *z*-score < −1.6. Only tests used in the identification of MCI or ALSci were included. Between group statistics from the primary MANCOVA were Bonferroni corrected for multiple comparisons (*p* < 0.05), education was entered as a covariate and degrees of freedom were 1, 36. Independent sample t-tests were conducted for the GDS, BDI, and BDI_veg. GDS degrees of freedom, 32; BDI degrees of freedom, 27*.

To assure our control group consisted of truly healthy volunteers, *z*-scores from neuropsychological tests were examined for mild cognitive impairment (MCI). Using standard criteria established in the literature, controls were excluded for suspected MCI if they scored at or below the fifth percentile on one or more cognitive test (Royall et al., 2004; Aarsland et al., 2010). Five controls were excluded for MCI, resulting in 17 participants in the HC group.

Age and years of education were compared between groups using independent sample t-tests, which showed no age differences (*p* = 0.65) but significantly more years of education in the HC group [*t*(37) = −3.65, *p* < 0.01]. Therefore education was included as a covariate in the following statistical models.

## Results

All patients with ALS completed the ALS Functional Rating Scale, revised version (ALSFRS-r). The ALSFRS-r is a 12-question survey measuring physical functioning of patients with ALS and is a strong predictor of progression and survival (Kollewe et al., 2008). Limb, bulbar, and respiratory functioning are measured in this scale and the maximum total score is 48 (lower scores indicate decreased physical function). Mean ALSFRS-r score for this patient group was 37.2 (*SD* = 6.9). Additionally, all participants completed the Oldenfield handedness questionnaire and a head trauma questionnaire (Armon and Nelson, 2012). Demographic information is presented in Table 1.

### Cognitive impairment in the ALS patient group

Using the consensus criteria developed by Strong et al. (2009), individual normalized scores were examined for each patient to identify those with possible ALS*ci*. Scores below the fifth percentile, or falling below a *z*-score of −1.6, were flagged. Individuals whose scores fell below this cut-off on two or more distinct tests were identified as patients with ALS*ci*.

Examining *z*-scores from each of the tests within the neuropsychological battery, eight of the 22 patients (36.4%) with ALS were identified as having CI. This finding is consistent with previous research showing an estimated 30% of patients with comorbid CI (Massman et al., 1996; Lomen-Hoerth et al., 2003; Rippon et al., 2006; Gordon et al., 2010; Raaphorst et al., 2010; Grace et al., 2011). Five of these patients scored at or below the threshold on the Animal Naming test and seven scored at or below the threshold on the HVLT. Scores from the Animal Naming and the HVLT tests were also among the most impaired scores overall in the ALS group, including scores from patients who did not meet criteria for ALS*ci*. Table 2 indicates the percent of patients who performed below the fifth percentile on each measurement.

A MANCOVA was conducted to test the effect of diagnosis group on cognitive performance, which showed a significant difference between ALS patients and HCs, *F*(13, 24) = 4.10, *p* = 0.001, η^2^ = 0.69. There was a specific significant difference between groups on Faces I, Faces II, COWAT, and Animal Naming. These statistics are shown in Table 2. The overall effect of education was not significant, *F*(13, 24) = 1.60, *p* = 0.15, η^2^ = 0.46, however education had an exclusive effect on the BNT, *F*(1, 36) = 7.04, *p* = 0.012.

Because verbal fluency performance can be confounded by speech production impairment in patients with ALS (Abrahams et al., 2004), we examined speed (in seconds) from Oral Trails-Making Test A between groups. An independent sample *t*-test showed that verbal speed differences approached significance, *t*(37) = 1.86, *p* = 0.07. Therefore a second MANCOVA was performed to evaluate the effect of diagnosis (ALS and HC) and verbal production speed (Oral Trails A seconds) on verbal fluency (COWAT and Animal Naming) performance. There was no main effect of verbal speed, *F*(2, 34) = 1.56, *p* = 0.22, η^2^ = 0.08 and no between-subjects effects of verbal speed on the Animal Naming test, *F*(1, 35) = 3.07, *p* = 0.09, or COWAT, *F*(1, 33) = 1.53, *p* = 0.22. There was still a main effect of diagnosis, *F*(2, 34) = 5.63, *p* < 0.01. These results suggest that differences in verbal fluency between groups were not confounded by verbal production speed.

### Physical symptoms of ALS did not have an effect on cognitive performance

#### Physical dysfunction did not predict global cognitive summary scores

The severity of physical dysfunction in patients with ALS may influence cognitive test performance. Some studies found that physical symptoms affect cognition differentially, specifically that those with more severe bulbar symptoms are more likely to have CI (Gordon et al., 2011). To test this and the influence of physical functioning on cognition, bulbar (questions 1–3) and limb (questions 4–9) functioning were derived from ALSFRS-r subscores. These were entered into a linear regression analysis with overall cognitive performance as the outcome variable. A cognitive performance summary score was calculated for each individual by taking the summation of *z*-scores from each of the cognitive test measure (*M* = −0.32, SD = 6.03). This model was not significant, *F*(2, 19) = 1.50, *p* = 0.25, *R^2^* = 0.05. Bivariate correlations (Pearson’s *r*) between physical symptoms and overall cognitive performance were also not significant: bulbar *p* = 0.45, limb *p* = 0.12.

#### Vegetative symptoms did not influence cognitive test scores

Another possible confound in a patient’s cognitive status could be attributed to vegetative symptoms common in patients with ALS. We therefore created a subscore of vegetative symptoms measured within the BDI, and examined the effect of these symptoms on cognitive test performance between patients and controls. Five questions from the BDI were used, described previously. A MANCOVA was conducted to test the hypothesis that vegetative symptoms may extraneously influence cognitive test performance. Diagnosis, education, and vegetative symptom subscores were entered as independent variables. Because patients are more likely to be affected by these symptoms due to physical weakness, we predicted that patients with ALS would be more negatively affected compared to HCs; therefore an interaction term between diagnosis group and vegetative symptoms was also included in this model.

This analysis showed no main effect for vegetative symptoms on cognitive performance, *F*(13, 12) = 1.31, *p* = 0.33, η^2^ = 0.59. However, vegetative symptoms did influence test performance on the Digit Span backward scores between groups, *F*(1, 24) = 5.25, *p* = 0.03, η^2^ = 0.18. Even after accounting for the possible effect of vegetative symptoms on cognitive performance, there was still a main effect of diagnosis group, *F*(13, 12) = 3.19, *p* = 0.03, η^2^ = 0.78. As in the original analysis, there was no effect of education, *F*(13, 12) = 1.06, *p* = 0.46, η^2^ = 0.54. Also, there was no interaction effect between vegetative symptoms and diagnosis group, *F*(13, 12) = 0.59, *p* = 0.82, η^2^ = 0.39.

Because only 14 patients completed the BDI, *t*-tests were conducted to test for demographic variable differences between patients with and without BDI data, and a MANOVA was conducted to test for a main effect on cognitive test performance. Age, education, disease duration, and progression rate (PR) did not differ between groups. There were also no differences between cognitive test scores. ALSFRS-r scores however approached significance, *t*(20) = 2.04, *p* = 0.06. The group who completed the BDI had a mean ALSFRS-r score of 39 (SD = 4.46) whereas those without had a mean ALSFRS-r score of 34 (SD = 8.98). Given the small sample size, it is possible that there was not an effect for vegetative symptoms on cognition because patients in this analysis were physically higher functioning. However, this importantly shows that patients with less disease severity still have overall poorer cognitive performance than HCs, independent of the effects of vegetative symptoms.

### Depressive symptoms in ALS in relation to disease severity and cognition

#### Patients with ALS were not more clinically depressed than HCs

We tested the hypothesis that patients with ALS were more depressed than the HC group by conducting independent sample *t*-tests using scores from the GDS. ALS patient mean scores were higher on the GDS than the HC group (see Table 2). Although these differences were statistically significant, the mean scores did not fall within clinical range for depression. When examining individual depression scores, two participants in each group met clinical criteria for possible mild depression as measured by the GDS. Vegetative symptoms, measured from the BDI, reported by patients were not different than the HC group.

#### Depressive scores correlated with progression rate (PR) and limb dysfunction

The fourth major goal of this study was to determine if depressive symptoms are related to disease severity in ALS. A correlation analysis using Pearson’s *r*-coefficient was conducted comparing GDS scores with PR. PR was calculated using the equation:

$\left(\text{PR}\right)=\frac{\left(48-\text{ALSFRS}-r\text{ Patients Score}\right)}{\text{Months Since Symptoms Onset}}$(Kollewe et al., 2008)

**Figure 1:**
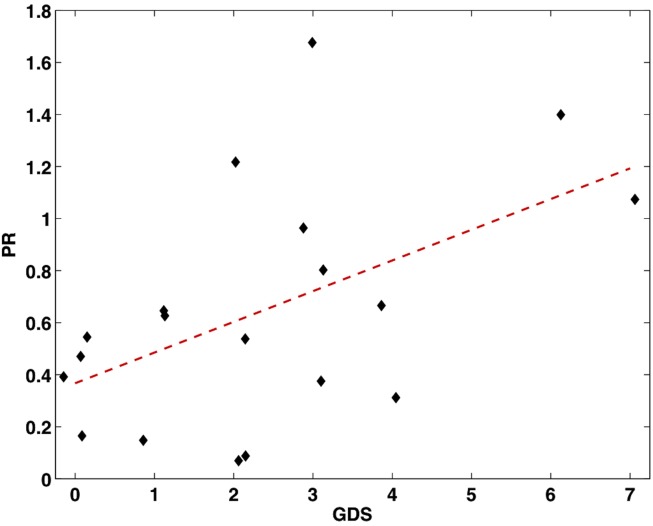
**Scatter plot showing the significant correlation between increased depressive scores from the GDS and faster progression rate (calculated by the PR equation, *n* = 19)**.

**Figure 2:**
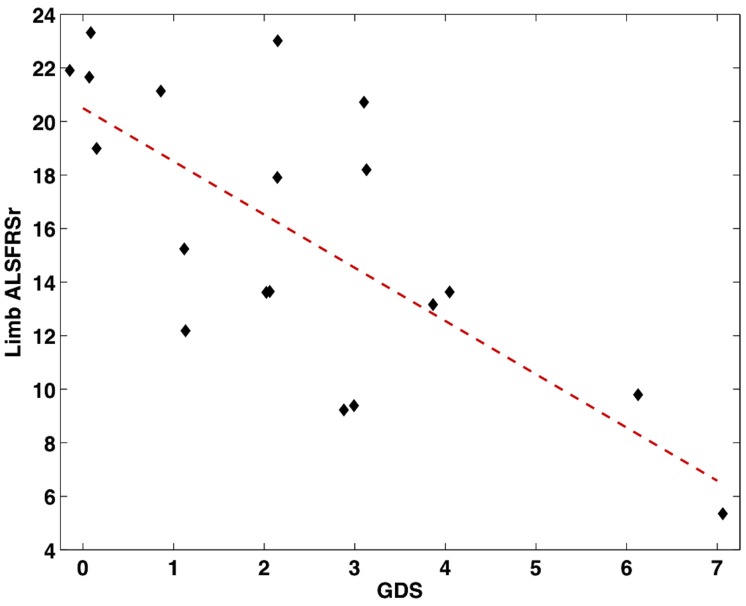
**Scatter plot showing the significant correlation between increased depressive scores from the GDS and decreased limb function measured from the ALSFRS-r (*n* = 19)**.

This correlation was significant, *r* = 0.52, *p* = 0.02 (see Figure 1). To further examine this relationship, the ALSFRS-r was divided into three subscores measuring bulbar (questions 1–3), limb (questions 4–9), and respiratory (questions 10–12) impairment. Of these models, depression scores negatively correlated with limb dysfunction, *r* = −0.72, *p* < 0.001 (Figure 2). Depression scores did not correlate with bulbar (*p* = 0.60) or respiratory symptoms (*p* = 0.49), however it should be noted that the range of bulbar and respiratory symptoms in our group was small.

#### Depressive symptoms affect cognitive test performance

The final MANCOVA in this study was performed to test the hypothesis that depressive symptoms have an influence on cognitive test performance differentially in patients with ALS. Depression may negatively impact cognition, and we aimed to answer whether ALS patients are more at risk for CI if they have comorbid depressive symptoms. Therefore, an interaction variable between GDS scores and diagnosis group and main effect variables of diagnosis group, GDS scores, and education were entered into the MANCOVA to test for effects on cognitive test measurements from the neuropsychological battery.

In this MANCOVA, there was a significant interaction effect between GDS scores and diagnosis group, *F*(13, 17) = 2.90, *p* = 0.02, η^2^ = 0.69. As expected, there were significant main effects of depressive symptoms, *F*(13, 17) = 3.34, *p* = 0.01, η^2^ = 0.72 and diagnosis group, *F*(13, 17) = 4.51, *p* < 0.01, η^2^ = 0.78. Examining the univariate effects on each cognitive test separately, there was interestingly not a significant main effect of depression on any single cognitive test, although the HVLT delayed recall scores approached significance, *p* = 0.07. There also was not a significant univariate effect of the interaction variable on any single cognitive test. Slopes of univariate analyses were examined to assess which group was more affected by depression on each cognitive test individually. Patients were more negatively affected by depressive symptoms on six of the cognitive test measurements (HVLT delayed recall, Faces II delayed recall, Oral Trails ratio, Animal Naming test, JOLO, and BNT), whereas the HCs were more negatively affected on five of the cognitive test measurements (MMSE, HVLT immediate recall and discrimination, Faces I immediate recall, and COWAT). This indicates there was not a specific pattern of which group was overall more affected by depressive symptoms, rather that the groups were indeed affected differently on individual cognitive domains. For example, patients appear more affected by depressive symptoms on tests of delayed recall whereas HCs appear more affected on tests of immediate recall.

## Discussion

There has been a recent push toward psychological intervention for patients with ALS in order to improve overall quality of life (Pagnini et al., 2012). Interventions would be better streamlined if risk factors for psychological distress were identified early, including risks for cognitive dysfunction and depression. In this study, we analyzed cognitive test performance between patients with ALS and a healthy control population. Importantly, we assessed the influence of physical and depressive symptoms on cognitive functioning in ALS, which may lead to better screening of individuals possibly at risk of having ALS*ci*. We also examined depressive symptoms in patients with ALS, and assessed if these depressive symptoms are related to disease progression or the manifestation of physical dysfunction specific to the disease process.

### Cognitive performance differences between patients with ALS and healthy controls

Results from our study corroborate previous findings, supporting cognitive performance differences between patients with ALS and HCs (Grossman et al., 2007; Strong et al., 2009). Overall, patients performed more poorly on cognitive testing than HCs, with specific deficits in executive function (verbal fluency) and visual recognition memory. Among patients identified as having ALS*ci*, the majority had impaired scores in semantic fluency and verbal learning and memory. Poorer performance in verbal fluency was not confounded by speech production speed. These findings are consistent with the literature, as verbal fluency remains the most sensitive to ALS cognitive dysfunction, and many others have noted impairment in memory and learning (Massman et al., 1996; Strong et al., 1996, 1999; Abrahams et al., 1997, 2004; Hanagasi et al., 2002; Grossman et al., 2007; Christidi et al., 2012). Utilizing neuropsychological tests that measure these cognitive domains would be most beneficial to screen for cognitive decline in a multidisciplinary ALS clinic setting, and have already been implemented in some clinics (Flaherty-Craig et al., 2009, 2011; Woolley et al., 2010).

### The effect of primary and secondary physical symptoms on cognition in ALS

Results did not support a unique contribution of limb or bulbar function on cognition. There has been no evidence that limb onset or symptoms are related to CI, and our results support previous research that found no specific effect of bulbar symptoms (Rippon et al., 2006; Gordon et al., 2010; Rusina et al., 2010). However, research on this is not consistent, and several studies have indicated that those with bulbar onset are more likely to be cognitively impaired (Lomen-Hoerth et al., 2003; Gordon et al., 2011). It may be possible that higher reports of CI in these patients are partly due to speech-motor impairment confounds, as a recent meta-analysis of 554 non-demented individuals with ALS did not find a relationship between CI and bulbar onset ALS (Raaphorst et al., 2010). Although we did not find an effect of bulbar symptoms on cognitive function, the number of patients with bulbar onset ALS were disproportionally less than those with limb onset, therefore our study was limited in that we could not conduct a comparative analysis between onset subtypes. Additionally, there was a small range of scores for bulbar symptoms, as measured by questions 1–3 in the ALSFRS-r (see Table 1), which may have contributed to our null result. Regardless, the involvement of bulbar symptoms on cognition, if any, should be further investigated to establish consensus.

One of our primary goals was to test whether vegetative symptoms confound or mask the interpretation of CI in ALS. Our results indicate, however, that these symptoms did not contribute to cognitive performance differences between patients with ALS and HCs. Although against our hypothesis, these results suggest that secondary physical symptoms, such as fatigue, collectively do not interfere significantly with test performance in patients with ALS. This finding was from a subgroup of patients in our study with overall high physical functioning, suggesting that CI manifests very early in the disease process. This finding supports ALS*ci* as a true variant of the disease, and increases the importance of understanding CI in ALS.

### Relationship between depressive symptoms, physical symptoms, and cognition in ALS

Depression contributes to decreased quality of life in patients with ALS (Tramonti et al., 2012), however our study indicated that as a group patients with ALS were no more likely to be depressed than a HC population. Although these results are consistent with recent reports (Averill et al., 2007; Atassi et al., 2011) that claim prevalence rates of depression are low in ALS, our study was limited by a small sample size and was not representative of a valid prevalence-rate study. Importantly, we found that increased depressive symptoms were associated with both disease progression and the severity of limb dysfunction. Severe global physical dysfunction has been shown to correlate with clinical depression (Oh et al., 2012), and now this effect has been demonstrated in a physically high functioning group without clinical depression (and using the revised version of the ALSFRS). Additionally, this relationship was most sensitive to limb dysfunction, suggesting those with more severe limb impairment may be at most risk. Given these findings, in conjunction with evidence for increased mortality risk in those with psychological distress (McDonald et al., 1994), future research on depression treatment and its association with survival should be emphasized.

Another important finding from our study indicates that depressive symptoms had an effect on cognitive performance. Although novel in the ALS literature, the relationship between depression and cognition has been demonstrated among other diseases (Diamond et al., 2008), and it has even been suggested that depression treatment could lead to improved cognitive performance (Sassoon et al., 2012). Likewise, a recent meta-analysis found that cognition can predict depression status, which could be targeted to increase treatment efficacy for depression (Phillips et al., 2010). Although there are no reports of depression treatment improving cognitive functioning or vise versa in ALS, these could potentially become important avenues for future translational research.

### Limitations and conclusions

#### Limitations

A major limitation in our study is that we were not able to test other factors that have been found to influence cognitive performance in patients with ALS. For example, Kim et al. (2007) found that patients with reduced forced vital capacity (FCV) performed significantly worse on tasks measuring memory retention/retrieval and verbal fluency than patients with normal vital capacity. Moreover, it has been suggested that impaired cognition might be reversible to some degree with increased vital capacity (Kim et al., 2007; Strutt et al., 2012). We suspect that patients in our study did not have impaired FVC for two reasons: they were enrolled in an MRI study and therefore able to lay flat on their back for more than 1 hour without respiratory distress, and scored near maximum on the ALSFRS-r questions measuring respiratory symptoms. Regardless, we did not explicitly acquire FVC measurements and were therefore unable to take into account this possible confound in our data.

Similarly, pseudobulbar affect is another symptom that may have a differential effect on cognition, and previous research has suggested that those with this symptom may be more at risk for CI (Abrahams et al., 1997). Again, we did not measure pseudobulbar affect symptoms and are therefore unable to account for this possible influence on cognition.

Our study also lacks a causal interpretation. We found that depressive symptoms and cognitive performance are related, but because we were unable to manipulate depressive symptoms or cognitive performance we were unable to identify the exact direction of this relationship. Additionally, our study showed that depressive symptoms are related to disease progression and limb dysfunction, yet it cannot be concluded whether increased depressive symptoms caused or directly influenced disease state. It is suggested that future studies examine depression and cognition intervention in order to identify a directional change in either disease state or cognitive performance.

Another major limitation in our study is the small sample size of groups. It is common for studies examining cognitive performance to include more than 100 participants, yet our study only compared 22 patients to 17 HCs. This is often a limitation in many rare disease population studies, however, and is especially common in the ALS literature. Future research would benefit from multicenter pooling of data, as suggested by Turner et al. (2011), in order to acquire larger sample sizes and gain higher statistical power.

In line with our small sample size, our analysis examining the effect of vegetative symptoms on cognition between groups was especially limited because only 14 patients completed the BDI. Those who completed the BDI were less severe in their disease symptoms. This analysis cannot therefore be generalized to the whole group in our study. However, this importantly shows that patients with less disease severity still have overall poorer cognitive performance than HCs, strengthening the finding that CI is a true symptom of ALS. The role of vegetative symptoms should be further explored, including larger sample sizes and patients with varying degrees of disease severity.

#### Conclusions

Overall, this study supported previous findings that CI is a symptom in approximately 35% of patients with ALS, and that patients overall perform worse than HCs in tests of executive dysfunction and learning and memory. Cognitive performance differences remained after accounting for variables including vegetative symptoms, physical dysfunction, and depressive symptoms. Additionally, we report that increased depressive symptoms are related to faster disease progression and greater limb dysfunction in patients with ALS. We therefore suggest that future research investigate the effects of depression therapy in patient care.

Although CI can significantly contribute to decreased quality of life for patients with ALS, research in this area is limited and often lacks a translational emphasis. Importantly, there is currently no treatment for patients who suffer from CI in ALS. Research though has highlighted several avenues to pursue in investigating CI intervention, including improving FVC (Kim et al., 2007; Strutt et al., 2012), treating pseudobulbar affect (Abrahams et al., 1997), and now therapy for depressive symptoms. Conversely, CI treatment may also improve depressive symptoms. We emphasize that future research focus on effective psychological or clinical interventions that may improve quality of life caused by CI or depression, and that these interventions be applied in future multidisciplinary ALS clinics.

## Conflict of Interest Statement

The authors declare that the research was conducted in the absence of any commercial or financial relationships that could be construed as a potential conflict of interest.
